# Cell behavior of the highly sticky bacterium *Acinetobacter* sp. Tol 5 during adhesion in laminar flows

**DOI:** 10.1038/s41598-018-26699-5

**Published:** 2018-05-29

**Authors:** Yoshihide Furuichi, Keita Iwasaki, Katsutoshi Hori

**Affiliations:** Department of Biotechnology, Graduate School of Engineering, Nagoya University, Furo-cho, Chikusa-ku, Nagoya, 464-8603 Japan

## Abstract

It is important to characterize how medically, industrially, or environmentally important bacteria adhere to surfaces in liquid flows in order to control their cell adhesion and subsequent biofilm formation. *Acinetobacter* sp. Tol 5 is a remarkably sticky bacterium that autoagglutinates through the adhesive nanofiber protein AtaA, which is applicable to cell immobilization in bioprocesses. In this study, the adhesion and behavior of Tol 5 cells in laminar flows were investigated using flow cell systems. Tol 5 cells autoagglutinated through AtaA and formed cell clumps during flowing. The cell clumps rather than single cells went downward due to gravity and adhered to the bottom surface. Under appropriate shear stress, a twin vortex was caused by a separated flow generated at the rear of the pre-immobilized cell clumps and carried the small cell clumps to this location, resulting in their stacking there. The rearward immobilized cell clumps developed into a large, stable aggregate with a streamlined shape, independent of cell growth. Cell clumps hardly ever developed under weak shear stress that could not generate a twin vortex and were broken up under excessively strong shear stress. These cell behaviors including the importance of clumping are interesting features in the bacterial adhesion processes.

## Introduction

Most bacteria initially adhere to surfaces, subsequently make microcolonies, and finally develop biofilms. In many cases, these steps occur and proceed in a liquid flow and are significantly affected by shear stress^1^. Many researchers have investigated bacterial cell adhesion or biofilm development under a laminar flow using flow systems. A liquid flow can affect microbial habitats by supplying nutrient, flushing out signaling molecules, and generating detachment forces. A liquid flow washes away quorum sensing autoinducers and represses quorum sensing, which is a chemical communication process for bacteria to coordinate gene expression in biofilms^2,3^. Chemical or enzymatic treatments which can alter the cohesion of bacterial biofilm change the ability to remove biofilms^4^. A strong flow, even laminar flow, can cause the detachment of bacterial cells from surfaces and the breakage of biofilms^5^. On the other hand, the adhesiveness of *Escherichia coli* to surfaces is enhanced through a conformational change of FimH under conditions of increased shear stress^6–8^. For *Pseudomonas aeruginosa*, the residence time of adhesion increases with increasing shear stress^9^. *Borrelia burgdorferi* and *Staphylococcus aureus* show shear-dependent increase in adhesion to endothelial cells with the bacterial adhesins, BBK32 or von Willebrand factor-binding protein^10–12^. *Bartonella henselae*, *Bartonella quintana*, and *Yersinia enterocolitica*, pathogenic bacteria that have trimeric autotransporter adhesin (TAA) for cell adherence, exhibit the adherence that appear more significant under dynamic conditions than static conditions^13^. These bacterial adhesive behaviors in response to shear stress vary among bacterial strains due to the diversity of their cell surface components or appendages. It is important to characterize how medically, industrially, or environmentally important bacteria adhere to surfaces in liquid flows in order to control their cell adhesion and subsequent biofilm formation. Furthermore, little has been discussed about the behavior of the cells with autoagglutinating nature during cell adhesion and biofilm development in flows; single cells adhere and grow to form a biofilm by a theory generally accepted for biofilm development^1^ or cell clumps formed by the autoagglutinating nature rather than single cells adhere to surfaces.

A non-pathogenic Gram-negative bacterium, *Acinetobacter* sp. Tol 5, was previously isolated from a biofiltration process^14^. This bacterium shows high adhesiveness to various abiotic surfaces from hydrophobic plastics to hydrophilic glass and stainless steel, and also demonstrates autoagglutination through its peritrichate fiber protein AtaA^15–17^. AtaA is a member of the TAA family, which contains proteins that are usually involved in bacterial adhesion to host cells and extracellular matrix proteins such as collagen and fibronectin, as well as in autoagglutination, colonization, biofilm formation, and serum resistance^18–23^. AtaA mediates the nonspecific, high adhesiveness to various abiotic surfaces mentioned above. This adhesive property can be conferred to originally non-adhesive bacteria by transformation with *ataA* and are applicable to cell immobilization in bioprocesses^24–26^. However, the behavior of Tol 5 cells in flows under the effect of shear stress has not yet been studied. In this study, the cell behavior of this sticky bacterial strain in laminar flows and the effect of shear stress on its cell adhesion were investigated.

## Materials and Methods

### Preparation of bacterial cells

The bacterial strains used in this study were *Acinetobacter* sp. Tol 5 wild-type (WT), its unmarked Δ*ataA* mutant Tol 5 4140 (Δ*ataA*)^27^, the Δ*ataA* mutant harboring pmCherry (Δ*ataA* (pmCherry)), *Acinetobacter baylyi* ADP1^28^, and its derivative strains harboring pARP3 (ADP1 (pARP3)) or pAtaA (ADP1 (pAtaA))^15^. The strain Δ*ataA* (pmCherry) was created in this study. The plasmids and the primers used for this purpose were listed in Supplementary Tables S1 and S2, respectively. To construct pmCherry, pHGE-P*tac*-GFP^29^ was digested with *Age* I and the linearized plasmid was re-circularized by self-ligation, generating pHGE-P*tac*-Δ*lacI-*GFP. The DNA fragment containing *Peredox-mCherry* was PCR-amplified from pRsetB-His7tag-Peredox-mCherry (Addgene plasmid 32382) using the primers, IF-Peredox-F and IF-Peredox-R. The PCR amplicon was cloned into the *Eco* RI site in pHGE-P*tac*-Δ*lacI-*GFP using In-Fusion HD Cloning Kit (Takara Bio, Shiga, Japan), generating pHGE-P*tac*-Δ*lacI-*Peredox-mCherry. To remove the *peredox* gene, inverse PCR was performed using the primers, Inverse-delta-Peredox-F and Inverse-delta-Peredox-R, and the PCR amplicon was digested with *Dpn* I and self-ligated to generate pHGE-P*tac*-Δ*lacI*-mCherry. Finally, the *mCherry* gene fragment was PCR-amplified using the primers, HiFi-mCherry-F and HiFi-mCherry-R, and cloned into the *Sal* I and *Xba* I site in pARP3^15^ using NEBuilder HiFi DNA Assembly Master Mix (New England BioLabs, Ipswich, MA). This plasmid was used for the transformation of Tol 5 Δ*ataA*, generating Δ*ataA* (pmCherry).

Bacterial cells were grown in Luria-Bertani (LB) medium for 12 h with shaking at 28 °C for Tol 5 WT and its derivatives or at 30 °C for ADP1 derivatives. Ampicillin (100 µg mL^−1^) and gentamicin (10 µg mL^−1^) were supplemented when required. Arabinose was added to a final concentration of 0.5% (*w/v*) at the inoculation for the induction of *mCherry*-gene or *ataA*-gene expressions under the control of the *P*_*BAD*_ promoter on the plasmids during growth. Arabinose was also given to cells harboring a vector control, as the same manner with cells harboring an expression vector. The stable production of AtaA on the cell surface of the ADP1 (pAtaA) in the presence of arabinose was shown previously^15^. After growth, the cells were harvested by centrifugation at 3,000 × *g* for 10 min, washed with deionized water (dH_2_O), and resuspended in dH_2_O to an optical density at 660 nm (OD_660_) of 0.4. After breaking up the cell clumps by ultrasound sonication three times (output 4 for 5 s; Tomy Seiko, Tokyo, Japan), the cell suspension was diluted to an OD_660_ of 0.2 with BS-N medium containing no carbon or nitrogen sources^30^. The resting cell suspension was used for the analyses of adherence and behavior of the cells using flow cell systems in the absence of arabinose.

### Construction and operation of the flow cell systems

**Figure 1:**
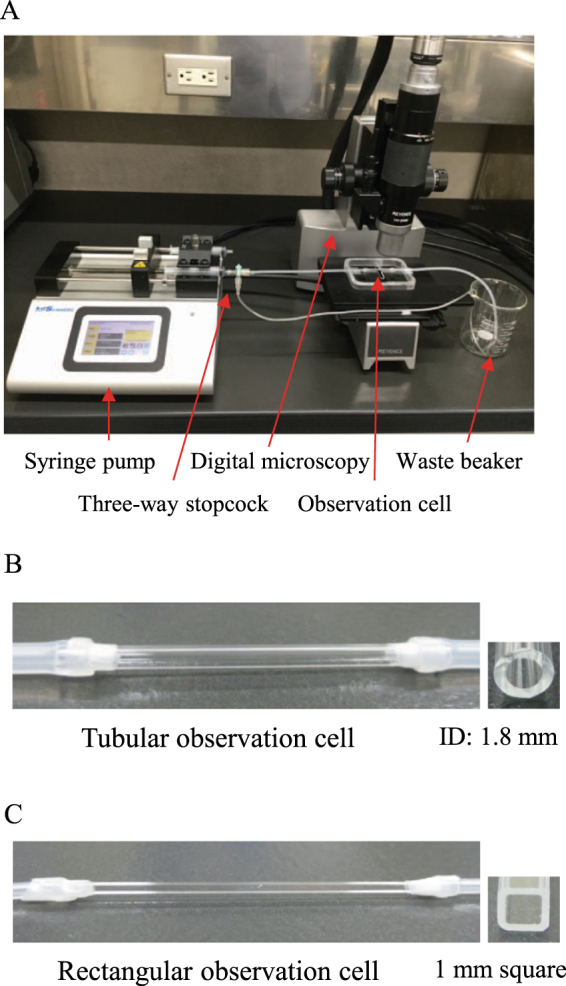
Construction of two different flow cell systems. (**A**) Overview of the flow cell system constructed in this study. (**B**) Tubular observation cell for the quantification of cell adhesion. (**C**) Rectangular observation cell for the observation of cell behavior in a liquid flow.

The basic structure of the flow cell systems constructed in this study was an observation cell of a glass tube that was connected to silicone tubes for the perfusion of cell suspension or liquids. To construct a tubular flow cell system, silicone tubes 300 mm in length, 2 mm in inner diameter (ID), and 4 mm in outer diameter (OD) were attached to both ends of a round glass tube 50 mm in length, 1.8 mm in ID, and 3 mm in OD (Iwaki, Tokyo, Japan), and their joints were sealed with paraffin film to avoid liquid spills. Likewise, to construct a rectangular flow cell system, silicone tubes 300 mm in length, 1 mm in ID, and 2 mm in OD were attached to both ends of a square glass tube 50 mm in length and 1 mm in every internal dimension (Vitrocom, Mountain Lakes, NJ), and their joints were also sealed with paraffin film. The inlet silicone tube was connected to a syringe pump (Legato 200; KD Scientific, Holliston, MA) via a three-way stopcock (Terumo, Tokyo, Japan) to perfuse liquids into the observation cell of the round or square glass tube. These flow cell systems are shown in Fig. 1. The procedure for the exchange of a liquid in the flow cell system with another liquid is depicted in Supplementary Figure S1, showing the exchange of BS-N medium with a crystal violet solution as an example.

Wall shear stress (τ) was calculated using equation (1):

1 $$\tau =\frac{8\mu U}{D}$$

where *μ*, *U*, and *D* are fluid viscosity, fluid velocity, and the diameter of the glass tube, respectively^31^. For the rectangular glass tube, the equivalent diameter (*De*) was used for the *D* value in equation (1), which was calculated according to equation (2):

2 $$De=\frac{4A}{P}$$

where *A* and *P* are the sectional area and wetted perimeter, respectively^32^. Reynolds number (*Re*) under each dynamic condition was calculated using equation (3):

3 $$Re=\frac{DU\rho }{\mu }$$

where *ρ* is liquid density^31^.

### Quantification of cell adherence and the thickness of cell clumps on a flow cell

To quantify the adherence of bacterial cells under laminar flow conditions, cells grown in LB medium were harvested by centrifugation at 3,000 × *g* for 10 min, washed with dH_2_O, and resuspended in dH_2_O to an OD_660_ of 0.4. After breaking up the cell clumps using ultrasound sonication as described above, 7 mL of the resting cell suspension diluted to an OD_660_ of 0.2 with BS-N medium was flowed at various velocities through the tubular flow cell system for cell adhesion. Thereafter, 7 mL BS-N medium was flowed to wash the glass tube at the same velocities as those used for cell adhesion, and subsequently, an aqueous solution of 0.1% crystal violet was flowed for 15 min at the same velocities to stain the still adhering bacterial cells. Finally, 7 mL BS-N medium was flowed again to wash the bacterial cells and remove the remaining dye. Thereafter, the glass tube was removed carefully from the flow cell system and the stain was dissolved in 200 µL of 2% SDS solution, and the absorbance at 590 nm (A_590_) was measured to quantify cell adherence.

To analyze the thickness of cell clumps, 7 mL of the cell suspension prepared by the same procedure above was flowed at various velocities through the rectangular flow cell system. After washing the glass tube with 7 mL BS-N medium, SYTO® 9 (Molecular Probes, Eugene, OR) diluted 1000 times with BS-N medium was flowed for 30 min at the same velocity to stain the cells, which were then washed with 7 mL BS-N medium. Thereafter, the glass tube was removed carefully from the flow cell system, sealed with paraffin film to avoid liquid spills, and subjected to confocal laser scanning microscopy (CLSM) (FV1000-D; Olympus, Tokyo, Japan) to obtain images of the bottom inner surface of the observation cells. The thickness of cell clumps was obtained from vertical x-z projections of the CLSM images.

### Direct observation of a flow cell system

A cell suspension was diluted with BS-N medium to an OD_660_ of 0.2, subjected to sonication to break up the cell clumps, and flowed through a rectangular flow cell system for 30 min at different velocities. Live images of the behavior of Tol 5 cells in liquid flows for their adhesion to the inner surface of the observation cell were recorded under a digital microscope (VHX-200; Keyence, Osaka, Japan). To quantify the areas occupied by the cells immobilized on the observation cell surface, photomicrographs obtained using the digital microscope were imported and their images were changed to binary images through ImageJ software^33^. To determine the areas occupied by immobilized cell clumps, the ‘Analyze Particles’ command of ImageJ software was used. The net increased areas where cells were immobilized during 10 min of flow under a shear stress of 7.57 mN m^−2^ were calculated from the occupied areas at 10, 20, and 30 min of the flow. The areas where cell clumps newly adhered to vacant spaces were manually designated on the images. The areas where the cell clumps stacked to the previously immobilized clumps were calculated by the net increased area minus the newly adhered area.

To visualize liquid flow in the flow cell system, 1 mL Tol 5 WT cell suspension (OD_660_ = 2.0) was mixed with 4 mL Δ*ataA* (pmCherry) cell suspension (OD_660_ = 2.5) and diluted with BS-N medium to a final volume of 10 mL. Then the mixture was subjected to sonication to break up the cell clumps of WT and flowed through a rectangular flow cell system under a shear stress of 7.57 mN m^−2^. The movement of Δ*ataA* (pmCherry) was traced under a fluorescence microscope (BZ-X700; Keyence, Osaka, Japan) at the frame rate of 0.2 frames per second. After recording the trace, the fluorescently visualized flow was merged with the cell clumps recorded in the same viewing under a bright-field condition using an image analysis software (BZ-H3A; Keyence, Osaka, Japan).

## Results

### Effects of shear stress on Tol 5 cell adherence

To examine the adhesion of Tol 5 resting cells under controlled shear stress conditions, two different flow cell systems equipped with either a round glass tube (tubular cell) or a square glass tube (rectangular cell) as an observation cell were constructed. They had an ID with a similar radius or dimension as the inlet and outlet tubes and had the same surface material (glass) surrounding the analysis area so that flow separation generated at the tube joints and heterogeneous adhesion arising from heterogeneity in surface materials could be theoretically ignored.

**Figure 2:**
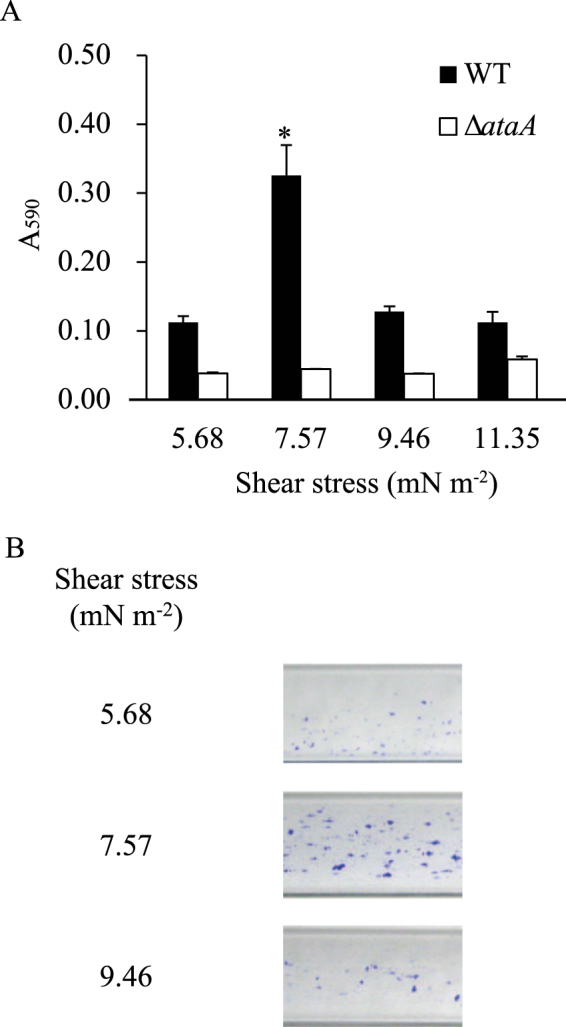
Adhesion of *Acinetobacter* sp. Tol 5 cells to a tubular observation cell at different shear stresses. (**A**) Quantification of Tol 5 WT (black) and Δ*ataA* mutant (white) cells that adhered during the flow of 7 mL of their suspension (OD_660_ = 0.2), shown by the absorbance at 590 nm of ethanol solutions containing crystal violet extracted from the cells after staining. Data are expressed as the mean ± standard error from three independent flows (n = 3). *P < 0.05. (**B**) Snapshots of the observation cells after staining adhering cells with crystal violet.

**Figure 3:**
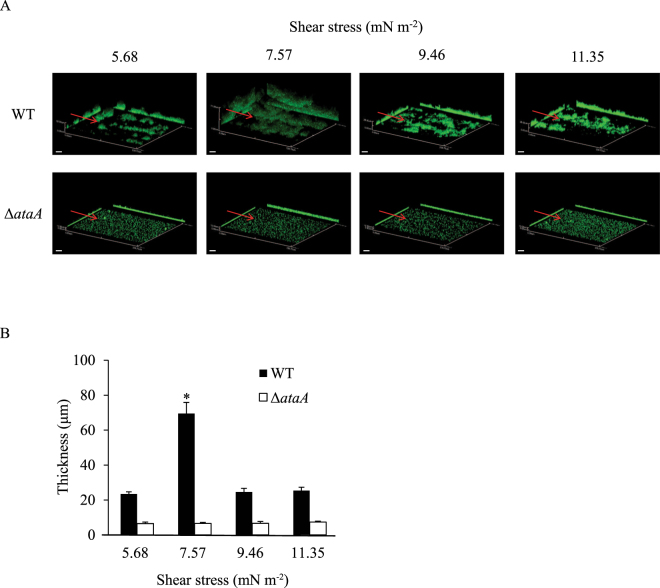
Quantification of the thickness of cell clumps formed by *Acinetobacter* sp. Tol 5 on a rectangular observation cell at different shear stresses. (**A**) Confocal laser scanning microscopy 3D-images with the x-z and y-z projections after flowing 7 mL of cell suspensions of Tol 5 WT and Δ*ataA* mutant cells (OD_660_ = 0.2). The red arrows indicate flow direction. Scale bar 20 µm. (**B**) Quantification data of the thickness of cell clumps formed by Tol 5 WT (black) and Δ*ataA* mutant (white) cells. Data are expressed as the mean ± standard error from three independent flows (n = 3). *P < 0.01.

First, a constant volume (7 mL) of bacterial cell suspensions of Tol 5 WT and the Δ*ataA* mutant at an OD_660_ of 0.2 were flowed through the tubular cell at different velocities (300, 400, 500, and 600 µL min^−1^), which produced shear stresses of 5.68, 7.57, 9.46, and 11.35 mN m^−2^, respectively, and bacterial cells that adhered to the inner surface of the tubular cell during the flow were quantified by staining with crystal violet. The Reynolds numbers (*Re*) from those flows were calculated to be 3.6, 4.8, 5.9, and 7.1, respectively, showing laminar flows. Measurement of the absorbance at 590 nm of the dye dissolved in ethanol solution revealed that significantly more WT cells adhered to the glass surface under the shear stress of 7.57 mN m^−2^ than under the other three shear stress conditions, which showed similar amounts of adhering cells (Fig. 2A). Conversely, the amount of the Δ*ataA* mutant cells that adhered was similar under all of the shear stress conditions examined and it was much smaller than that of WT cells, confirming that AtaA greatly contributes to the adhesion of Tol 5 cells in liquid flows. The adhesion of more cell clumps with larger sizes was observed at 7.57 mN m^−2^ than at the other shear stress conditions (Fig. 2B). Then, to investigate the thickness of the cell clumps immobilized onto a glass surface, the bacterial cells were flowed through the rectangular cell at 47.8, 63.8, 79.7, and 95.6 µL min^−1^, so as to produce the same shear stresses as those through the tubular cell, and cell adhesion was observed using CLSM (Fig. 3A). WT cells formed thick cell clumps only under the shear stress of 7.57 mN m^−2^ and thin cell clumps were formed under the other shear stress conditions (Fig. 3B). On the other hand, Δ*ataA* cells could not accumulate on the glass surfaces in each flow, confirming that AtaA is responsible for the clumping of Tol 5 cells.

Next, bacterial cell suspensions were flowed through the tubular cell at different velocities for a constant time period (40 min) so that the contact time of the suspensions to the flow cell surface was consistent between the conditions. Although the total volume flowed varied according to the velocity used in these flow conditions, the same results were obtained as those for when the flow volume was consistent; that is, the amount of WT cells that adhered to the glass surface was the largest under the shear stress of 7.57 mN m^−2^ (see Supplementary Figure S2). Therefore, the most preferable shear stress for the adhesion of Tol 5 cells was 7.57 mN m^−2^ in the range of shear stresses tested using the flow cell system constructed in this study regardless of the flow parameters, such as flow volume and contact time.

### Behavior of bacterial cells expressing the *ataA* gene during adhesion under laminar flows

**Figure 4:**
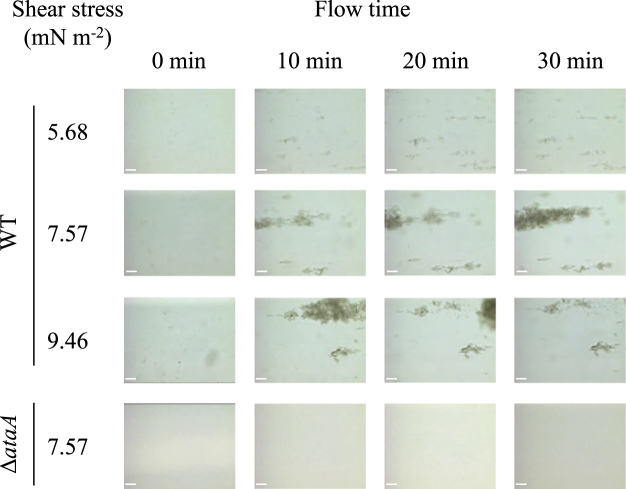
Snapshots of the adhesion process of *Acinetobacter* sp. Tol 5 WT cells to the bottom surfaces of rectangular observation cells at different shear stresses. Tol 5 cell suspension was flowed through the observation cell for 30 min at the shear stresses of 5.68, 7.57, and 9.46 mN m^-2^ respectively, and its bottom surface was observed by digital microscopy. The flow direction is from left to right in each panel. Scale bar 50 µm.

To reveal why more Tol 5 cells adhered under the shear stress of 7.57 mN m^−2^ than under the other conditions, the process of cell adhesion to the bottom surface of the rectangular cell was observed in flows with different shear stresses using a digital microscope. At 7.57 mN m^−2^, Tol 5 WT cell clumps adhered to the bottom surface, and these immobilized clumps gradually became larger, forming a streamlined shape (Fig. 4). At 5.68 mN m^−2^, small cell clumps adhered to the bottom surface, but few of them became larger. At 9.46 mN m^−2^, large cell clumps adhered and became larger, but eventually breakage of the clumps occurred. However, even at 7.57 mN m^−2^, hardly any Δ*ataA* mutant cells adhered to the flow cell surface. The development of cell clumps into streamlined shapes was also observed when cells of another bacterium expressing the *ataA* gene, *A. baylyi* ADP1 (pAtaA), were flowed at 7.57 mN m^−2^ for 40 min, whereas hardly any ADP1 cells transformed with the vector control pARP3 adhered to the surface, confirming that AtaA was responsible for the development of the immobilized cell clumps into this characteristic shape (see Supplementary Figure S3).

Interestingly, cell clumps flowing at 7.57 mN m^−2^ stacked at the rear of the clumps that were previously immobilized onto the bottom surface, developing into a streamlined shape (see Supplementary Movie S1). To reveal the mechanism of this developing pattern, the liquid flow conveying bacterial cells was visualized using non-autoagglutinating cells, Δ*ataA* (mCherry) cells, under a fluorescent microscope. It was observed that the flow was altered by an immobilized cell clump and made a turn to the rear of it (see Supplementary Movie S2). In addition, it revealed that a twin vortex generated by the flow turn to the rear of the immobilized cell clump contributes to its rearward development into the larger clumps under this shear stress (see Supplementary Movie S3).

**Figure 5:**
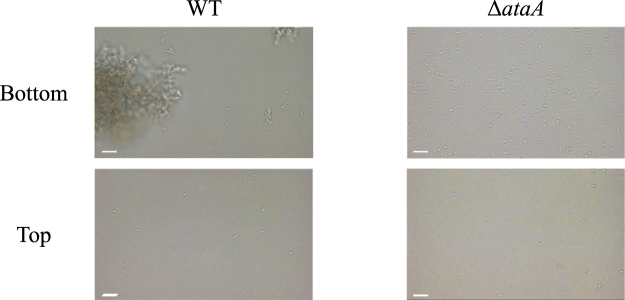
Adhesion of *Acinetobacter* sp. Tol 5 WT and Δ*ataA* cells to the top and bottom surfaces of a rectangular observation cell. The cell suspension of each strain was flowed through the observation cell for 30 min at a shear stress of 7.57 mN m^-2^, and the top and bottom surfaces of the cell were observed by digital microscopy. The flow direction is from left to right in each panel. Scale bar 10 µm.

To investigate the effect of gravity on cell adhesion to surfaces in a laminar flow, bacterial cell adherence was compared between the top and bottom surfaces of the rectangular cell under the shear stress of 7.57 mN m^−2^. To the bottom surface, WT cells formed large cell clumps and adhered, while single Δ*ataA* cells adhered without forming cell clumps (Fig. 5). To the top surface, however, a few single WT and Δ*ataA* cells but no WT cell clumps were observed to adhere, showing the great contribution of gravity to the adhesion of Tol 5 cells and their clumps in laminar flows.

### Contribution of cell clumps to Tol 5 cell adhesion to a surface in a laminar flow

**Figure 6:**
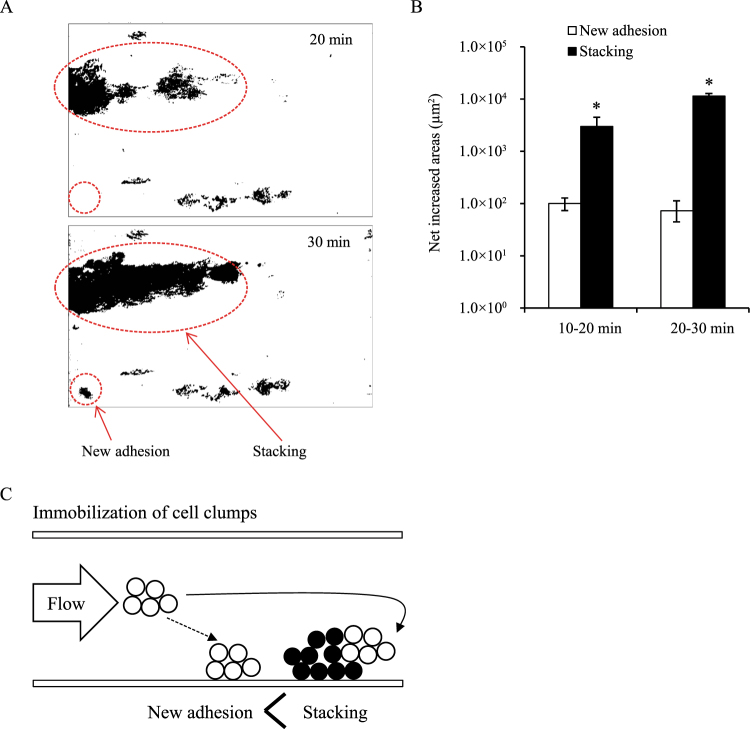
Comparison of the areas occupied by *Acinetobacter* sp. Tol 5 cells that were immobilized by stacking to pre-immobilized cell clumps with the cells newly adhering to vacant spaces. (**A**) Binary images converted from digital images obtained using a digital microscope. (**B**) Net increased areas occupied by the cell clumps calculated from the binary images using ImageJ. Data are represented as the mean ± standard error from three independent flows (n = 3). *P < 0.05. (**C**) Schematic of the immobilization of Tol 5 cell clumps through new adhesion and stacking in a flow.

The experimental results described above suggested that the formation of cell clumps is important for Tol 5 cells to adhere to the surface of the rectangular cell in a laminar flow. In support of this, a movie of the process of Tol 5 cell adhesion shows that the cell clumps grew larger during flowing and that cell clumps rather than single cells adhered to the bottom surface (see Supplementary Movie S4). Finally, the area occupied by cell clumps developing through the stacking of new clumps to the previously immobilized clumps was compared with that occupied by cell clumps that newly adhered to vacant spaces on the bottom surface (Fig. 6A). The former area was larger than the latter area (Fig. 6B). This revealed that cell clumps tend to stack on cell clumps that were previously immobilized to the surface rather than adhere to vacant spaces of the surface (Fig. 6C).

## Discussion

Many researchers have described the processes of bacterial adhesion and biofilm formation. As a rule, bacterial adhesion is the initial step in biofilm formation. Firstly, bacterial cells approach surfaces by motility or Brownian motion and adhere to the surfaces reversibly. Thereafter, the cells produce EPS, attain irreversible adhesion, and form a microcolony, which finally develops into a biofilm^34–36^. However, there are still unclear points about the behavior of cells during cell adhesion and biofilm development in liquid flows. Little is known regarding which is the prevalent mechanism among the following cell behaviors; single cells adhere and grow to form a microcolony and a biofilm, planktonic single cells newly take part in a biofilm, or cell clumps adhere to surfaces and/or preformed biofilms. Conversely, a uniform description of cell behavior seems to be difficult due to the variety of bacterial characteristics related to cell adhesion, such as motility, adhesiveness, autoagglutinating nature, and EPS production.

In this study, the cell behavior of *Acinetobacter* strains expressing the *ataA* gene, whose product protein mediates the noteworthy autoagglutinating nature and adhesiveness of these strains to various material surfaces, was observed using flow cell systems of our own making, which allowed quantification and observation of the cell adhesion, eliminating the effects of artificially generated flow separation and of heterogeneity in the material surface. The flow cell systems that have been commercialized or that are usually used are parallel plate chamber types composed of two transparent plates separated by spacers^37^ or multi-channel excavated base plate types that are made of polymethyl methacrylate and mounted with a coverslip using glue^38^. Researchers have used flow systems that are suitable for their purposes, utilizing commercialized, modified, or self-built ones. However, many of conventional flow cell systems generate a separated flow at the joint between the observation cells and the inlet and/or outlet tubes due to differences in the widths of the observation cells and the radii of the tubes, which affect the behavior and adhesion of bacterial cells^31^. Another important point for the quantification of the adherence of bacterial cells that show high adhesiveness is that the same material should be used for the surfaces surrounding the analysis area to avoid heterogeneous adhesion.

**Figure 7:**
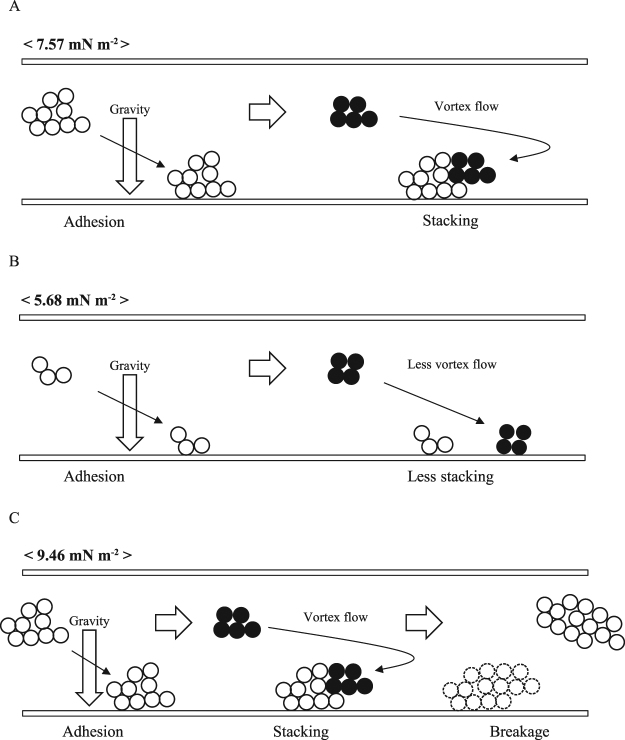
Representations of the behavior of Tol 5 cell clumps under laminar flow conditions. (**A**) Under appropriate shear stresses, such as 7.57 mN m^-2^, the cell clumps adhere to the bottom surface and are immobilized there, affected by gravity, and develop through the stacking of the cell clumps conveyed to the rear by the twin vortex. (**B**) Under lower shear stresses, flowing cells form smaller cell clumps, but hardly any of them stack onto the immobilized clumps due to the absence of the twin vortex. (**C**) Under higher shear stresses, the immobilized clumps develop as under appropriate shear stresses, but the clumps are eventually broken up by the high shear stress.

By using resting cells suspended in a carbon and nitrogen source-free medium, cell growth could be ignored. It was demonstrated that even without cell growth, cells expressing the *ataA* gene autoagglutinate, and then the formed cell clumps rather than single cells adhere to the bottom surfaces of the flow cell system and are immobilized there in laminar flows. The immobilized cell clumps develop through stacking of small cell clumps carried by the twin vortex flow to their rear area, independent of cell growth, under appropriate shear stresses, such as 7.57 mN m^−2^ (Fig. 7A). The adhesion of the cell clumps is clearly influenced by gravity and hardly any of the cells adhere to the top surface in spite of their high adhesiveness mediated by AtaA. Under lower shear stresses, flowing cells form smaller cell clumps and they are stacked ineffectively onto the immobilized clumps (Fig. 7B). Under higher shear stresses, immobilized cell clumps develop similarly, but the high shear stress eventually breaks up the immobilized clumps (Fig. 7C).

Hydrodynamic vortices, such as a twin vortex and Karman vortex, are caused by a separated flow at the rear of obstacles in liquid flows, depending on the Reynolds number (*Re*)^39^. Taneda reported that a twin vortex began to form at an *Re* of approximately 5, whereas no twin vortex was observed at an *Re* of 3.64^40^. Therefore, it is reasonable to hypothesize that a twin vortex develops at the rear of Tol 5 cell clumps at an *Re* of 4.8 and 5.9 under the shear stress of 7.57 mN m^−2^ and 9.46 mN m^−2^, respectively; the twin vortex guides relatively small cell clumps to the rear of larger clumps pre-immobilized onto the bottom surface and causes the cell clumps to be stacked there. Finally, at 7.57 mN m^−2^, they are deformed into a streamlined shape, which is a structurally stable topology in a shear flow^41^. However, at 9.46 mN m^−2^, the immobilized cell clumps are under considerable stress so that it is difficult for them to develop into a firm aggregate and they are eventually broken up. Conversely, at an *Re* of 3.6 under the shear stress of 5.68 mN m^−2^, *Re* is so small that no twin vortex is generated. Therefore, cell clumps have few chances to be conveyed to the rear of pre-immobilized cell clumps and to collide with them and adhere. In summary, Tol 5 cells autoagglutinate through AtaA and form small cell clumps before its single cells individually adhere in laminar flows. The cell clumps go to the bottom due to gravity, adhere to the bottom surface through AtaA, and are immobilized there. Under a condition of appropriate shear stress, a twin vortex is caused by separated flow generated at the rear of the immobilized cell clumps and conveys small cell clumps to the location of pre-immobilized cell clumps, resulting in the stacking of the cell clumps there. The immobilized cell clumps rearward develop into a large and stable aggregate, that is, a ‘biofilm-like structure’, with a streamlined shape under a shear flow, independent of cell growth. Investigation of the adhesion and behavior of Tol 5 cells in turbulence flows is the next step in our further study.

## Electronic supplementary material

Supplementary Information

Supplementary Movie S1

Supplementary Movie S2

Supplementary Movie S3

Supplementary Movie S4
