# Supplementary material for: Cell behavior of the highly sticky bacterium Acinetobacter sp. Tol 5 during adhesion in laminar flows
Source: Sci Rep. 2018 May 29;8:8285. doi: 10.1038/s41598-018-26699-5 (PMC5974025; doi:10.1038/s41598-018-26699-5)
Supplement: Supplementary file 1 — Supplementary Information [file 41598_2018_26699_MOESM1_ESM.docx]

Cell behavior of the highly sticky bacterium *Acinetobacter* sp. Tol 5 during adhesion in laminar flows

Yoshihide Furuichi, Keita Iwasaki, Katsutoshi Hori*

Department of Biotechnology, Graduate School of Engineering, Nagoya University, Furo-cho, Chikusa-ku, Nagoya 464-8603, Japan

**Supplemental material contents**

Table S1

Table S2

Figure S1

Figure S2

Figure S3

Movie S1

Movie S2

Movie S3

Movie S4

**Table S1.** Plasmids used in this study

| Plasmid | Description | Reference |
| --- | --- | --- |
| pHGE-P*tac*-GFP | Broad host expression vector, P*_tac_*, *lacI*, Km^r^ | Luo et al. 2013 |
| pHGE-P*tac*-Δ*lacI*-GFP | Δ*lacI* mutant of pHGE-P*tac*-GFP | This study |
| pRsetB-His7tag-Peredox-mCherry | Expression vector, T7, Amp^r^, *peredox-mCherry* | Hung et al. 2011 (Addgene 32382) |
| pHGE-P*tac*-Δ*lacI*-Peredox-mCherry | *gfp::peredox-mCherry* mutant of pHGE-P*tac*-Δ*lacI*-GFP | This study |
| pHGE-P*tac*-Δ*lacI*-mCherry | Δ*peredox* mutant of pHGE-P*tac*-Δ*lacI*-Peredox-mCherry | This study |
| pARP3 | *E.coli-Acinetobacter* shuttle expression vector, *araC*-P*_BAD_*, Amp^r^, Gm^r^ | Ishikawa et al. 2012 |
| pmCherry | pARP3 containing *mCherry* | This study |
| pAtaA | pARP3 containing *ataA* | Ishikawa et al. 2012 |

**Table S2.** Primers used in this study

| Primer | Sequence (5’→3’) |
| --- | --- |
| IF-Peredox-F | ACACAGGAGAGAATTCATGAAACATCACCATCATCATC |
| IF-Peredox-R | CGAGCTCCATGAATTGATGGCCCACTACAGCTTGG |
| Inverse-delta-Peredox-F | GGTCTGGTGGTATGGTGAGC |
| Inverse-delta-Peredox-R | GGATTCTCTCCTGTGTGAAATTG |
| HiFi-mCherry-F | CCCGTTTTTTTGGGCTAGCGAATTCACTTTAAGAAGGAGATATACATATGGTGAGCAAGGGCGAG |
| HiFi-mCherry-R | CCACCGCGGTGGCGGCCGCTCTAGAGCCATCAATTCATGGAGCTC |


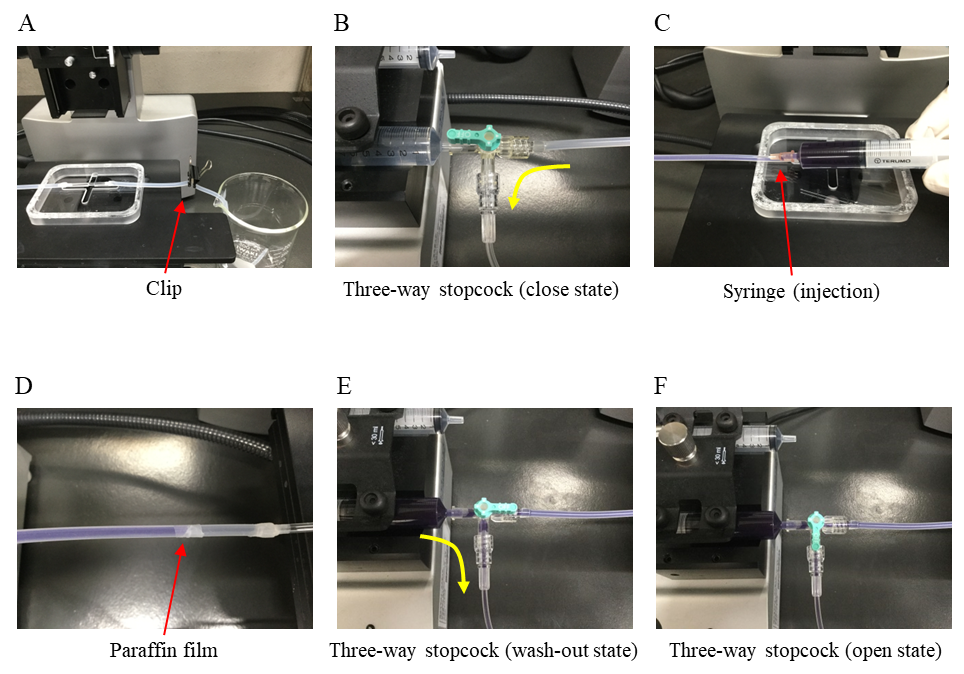


**Figure S1.** Procedure for liquid exchange in the flow cell system. (A) To exchange BS-N medium in the flow cell system, the outlet tube from the observation cell was clipped to prevent the liquid inside the glass tube from flowing out. Then, (B) a three-way stopcock was closed and (C) a crystal violet solution was injected into the inlet tube to flush out the BS-N medium through the path of the three-way stopcock indicated by the yellow arrow in (B). (D) The injection point was sealed with paraffin film to avoid liquid spills. (E) After switching the three-way stopcock to the wash-out position, the syringe containing the BS-N medium was replaced with another syringe containing the crystal violet solution, which was then used to flush out the BS-N medium remaining in the three-way stopcock in the direction indicated by the yellow arrow. Finally, (F) the three-way stopcock was opened and the clip was unfastened to return to the initial state. The flow direction of the syringe pump is from left to right in each panel.


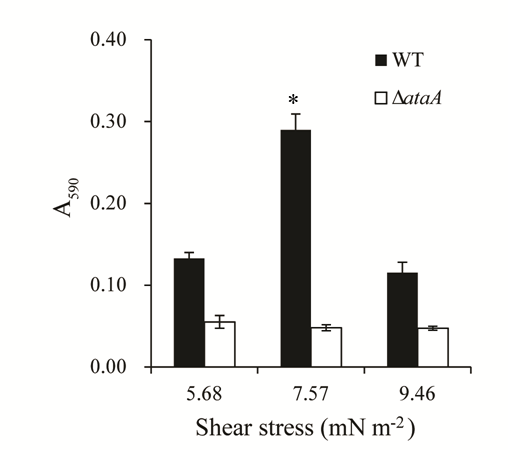


**Figure S2.** Adhesion of *Acinetobacter* sp. Tol 5 cells to a tubular observation cell at different shear stresses. Quantification of Tol 5 WT (black) and Δ*ataA* mutant (white) cells that adhered during flowing of their suspension (OD_660_ = 0.2) for 40 min, shown by the absorbance at 590 nm of ethanol solutions containing crystal violet extracted from the cells after staining the cells. Data are represented as the mean ± standard error from three independent flows (n = 3). *P < 0.05.


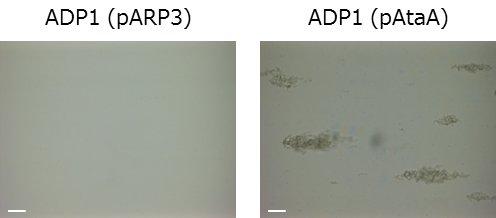


**Figure S3.** Appearance of the cell adhesion of *A. baylyi* ADP1 (pAtaA) producing AtaA or ADP1 (pARP3) (vector control) after flowing their suspension (OD_660_ = 0.2) for 40 min under the shear stress of 7.57 mN m^-2^. Scale bar 50 µm.

**Supplementary Movie S1.** Stacking of flowing cells at the rear of immobilized clumps.

**Supplementary Movie S2.** Visualization of altered flow.

**Supplementary Movie S3.** Observation of a twin vortex.

**Supplementary Movie S4.** Development of the size of flowing cell clumps during a flow.
